# Pyogenic Spondylodiscitis Following Nonspinal Cesarean Section

**DOI:** 10.7759/cureus.45966

**Published:** 2023-09-25

**Authors:** Amani Alshami, Banan Alkharat, Zeina Alwattar, Mhd Firas Safadi

**Affiliations:** 1 Rheumatology Department, Al-Badr International Hospital, Ibb, YEM; 2 Infectious Disease Department, Al-Mowasat University Hospital, Damascus University, Damascus, SYR; 3 Internal Medicine Department, Al Razi Hospital, Homs, SYR; 4 General and Visceral Surgery Department, Zeisigwaldkliniken Bethanien Chemnitz, Chemnitz, DEU

**Keywords:** lower back pain, lumbar pyogenic discitis, nonspinal cesarean section, cesarean section complications, cesarean section, postoperative back pain, postoperative pyogenic discitis, sacral discitis, vertebral osteomyelitis, pyogenic spondylodiscitis

## Abstract

Pyogenic spondylodiscitis is an uncommon bacterial infection of the intervertebral disc and the vertebral endplates. It usually affects elderly patients with comorbidities but may be also seen after surgical procedures in young patients, mostly after spinal interventions and genitourinary procedures. This article describes a rare case of pyogenic spondylodiscitis in a young female patient after a cesarean section without spinal anesthesia. The patient presented with a three-month history of lower back pain, and the inflammatory markers were elevated. The magnetic resonance imaging showed the involvement of the L5-S1 disc space and the adjacent vertebral bodies. The diagnosis was confirmed with needle aspiration of purulent material. The patient was treated with antibiotics for a total of six weeks. After a follow-up of about one year, the patient showed slight degenerative vertebral changes with no signs of residual infection. This case highlights the importance of the early recognition of pyogenic spondylodiscitis as one cause of postoperative back pain after urogenital procedures, even without spinal anesthesia. Only a few similar cases were reported in the literature.

## Introduction

Pyogenic spondylodiscitis is a bacterial infection of the vertebral column that involves the intervertebral disc and/or the adjacent vertebral endplates [1]. The course of the disease is usually subtle with back pain and elevated inflammatory markers. The imaging modalities, mainly magnetic resonance imaging (MRI), show local destructive changes, and the diagnosis can be confirmed with fluid aspiration and bacterial culture [2,3].

Most commonly, the disease affects elderly immune-compromised patients. It was also reported in young healthy patients after surgical procedures, mainly interventions on the genitourinary system as well as those performed under spinal anesthesia [4].

In this report, we present a case of a young female patient who presented with persisting lower back pain three months after a cesarean section under general anesthesia. Only a few cases of pyogenic spondylodiscitis are reported after cesarean section without spinal anesthesia.

## Case presentation

A 34-year-old woman presented to the rheumatology clinic with a three-month history of lower back pain. Her complaints started on the second day after a cesarean section under general anesthesia and showed no improvement since then. The indication for surgery was a previous cesarean section, and the patient had no postoperative complications.

The pain was localized to the lower back without radiation to the extremities. It was not related to the time of the day, but it was exacerbated by sitting. The patient tried to relieve her symptoms with nonsteroidal anti-inflammatory drugs (NSAIDs) with no obvious response. She denied fever, chills, or night sweating. No significant weight changes were noted since delivery. Apart from her cesarean section, her past medical, surgical, and familial history was nonsignificant.

On physical examination, the patient was afebrile with stable vital signs. Inspection of the back showed no skin abnormalities, but the vertebral percussion elicited tenderness over the lower lumbar vertebrae at the L4-S1 level. Lateral and anterior flexion of the lumbar spine was limited and painful. The straight leg test was negative bilaterally, and there were no neurological deficits.

Investigations

**Figure 1:**
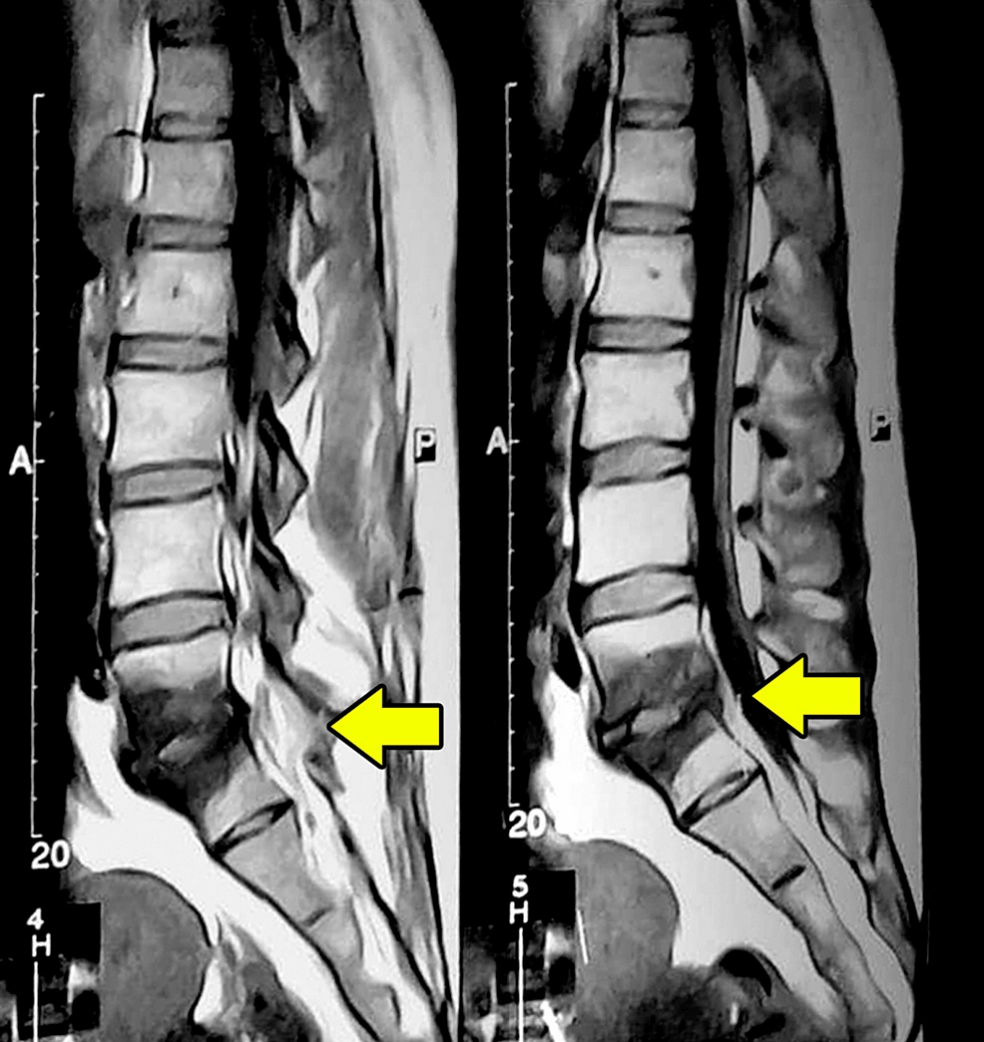
Pre-treatment MRI images. Two sagittal sections of the T1-weighted MRI on initial presentation showing involvement of the L5-S1 disc space and adjacent vertebral bodies with decreased signal intensity. No lesions are seen in the other disc spaces or vertebrae.

Laboratory investigations revealed mild anemia with a slight elevation of the erythrocyte sedimentation rate (ESR) and procalcitonin (Table 1). These results implied an infectious vertebral process and guided the next diagnostic steps. The skin tuberculin test and the bacterial blood cultures were negative. The imaging study of the lumbar spine using MRI showed signs of discitis at the L5-S1 level with involvement of the adjacent vertebral endplates (Figure 1). For further bacterial diagnosis, the patient underwent aspiration of the affected disk under computed tomography (CT) guidance, which yielded purulent fluid. Immediately after the aspiration, the patient reported symptomatic improvement.

**Table 1:** Laboratory results in serum. CRP, c-reactive protein; ELISA, enzyme-linked immunosorbent assay; ESR, erythrocyte sedimentation rate; PCT, Procalcitonin; SAT, standard agglutination test

Laboratory assay	Patient’s results	Reference range
Haemoglobin, g/L	11.4	12-16
White blood count, x10^9^/L	7.8	4.5-10.2
CRP, mg/L	2.77	0-6
ESR, mm/h	40 (elevated)	0-20 (first hour)
PCT, ng/dl	0.1 (elevated)	< 0.05
Blood urea nitrogen, mg/dl	13	13-43
Creatinine, mg/dl	0.52	0.5-2.0
Brucella SAT	Negative	Negative
Brucella serologic ELISA antibodies	Negative	Negative
Tuberculin skin test	Negative	Negative

Treatment

Based on the laboratory, imaging, and aspiration findings, we made the diagnosis of pyogenic spondylodiscitis and started empiric therapy with vancomycin, ceftazidime, and levofloxacin. This combination was recommended based on the bacterial data of postoperative infections in our institute with coverage of Pseudomonas and Enterobacteriaceae. Since the bacterial cultures were negative, we discharged the patient and proceeded with the same intravenous regimen in an outpatient setting for a total of two weeks. Afterward, we switched to intramuscular ceftazidime as well as oral linezolid and levofloxacin for one additional week. The oral therapy was then continued for three additional weeks.

Outcome and follow-up

**Figure 2:**
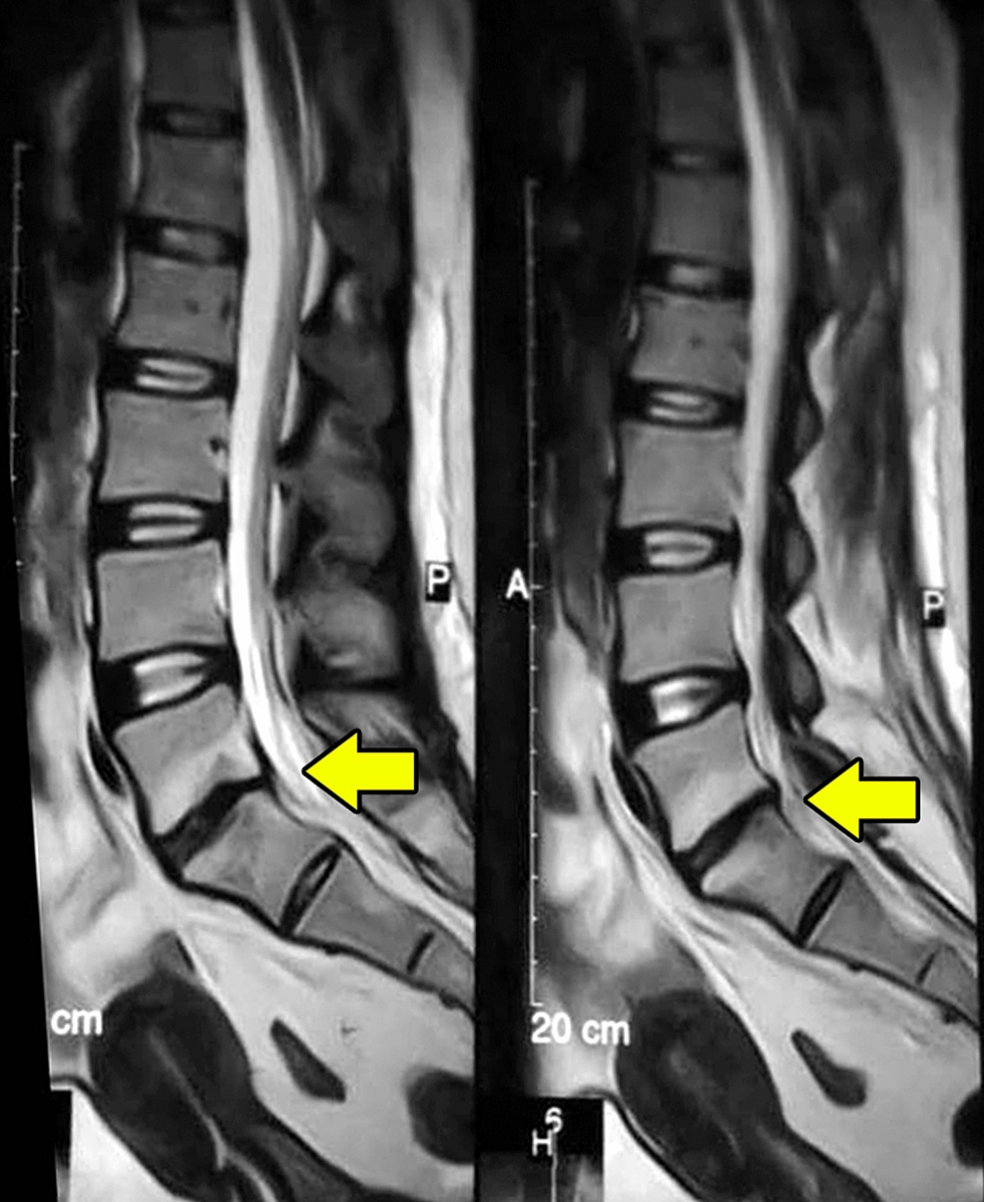
Post-treatment MRI images. Two sagittal sections of the T1-weighted MRI after six weeks of antibiotic treatment. The imaging showed complete resolution of the discitis with the presence of endplate irregularities at the L5-S1 level.

A follow-up MRI after six weeks showed resolution of the discitis with the presence of endplate irregularities (Figure 2). The therapy was associated with gradual resolution of pain, and the patient was able to resume her usual activities. The inflammatory markers returned to normal and the therapy was terminated at this point with a total duration of six weeks.

**Figure 3:**
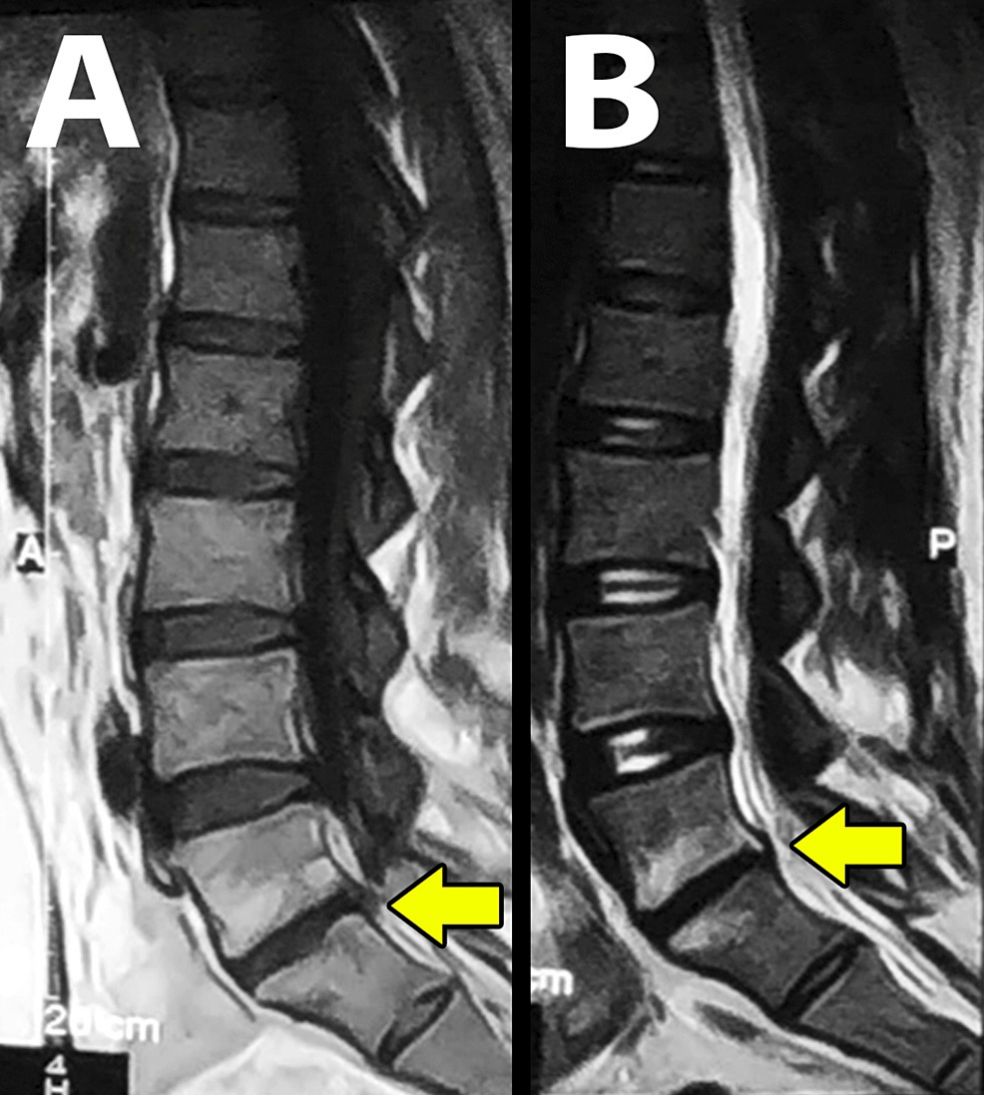
Follow-up MRI images. T1-weighted (A) and T2-weighted (B) MRI in the sagittal plane about one year after the treatment. The imaging showed relative L5-S1 disk space narrowing without inflammatory changes.

Eleven months later, the patient presented again with mechanical lower back pain in the same region. A control MRI showed narrowing in the disc space L5-S1 with an altered signal in the adjacent vertebral endplates. There was no disc herniation, spinal stenosis, bone marrow edema, or fluid collections around the disk (Figure 3). With the diagnosis of degenerative vertebral pain, the patient was treated with NSAIDs over many weeks and showed substantial improvement.

## Discussion

Pyogenic spondylodiscitis, also called pyogenic vertebral osteomyelitis, is an infection of the intervertebral disc and/or the adjacent vertebral endplates [1]. Recent studies showed an increased incidence of the disease from 2.9 to 5.4 per 100,000 [5]. Elderly patients with comorbidities such as diabetes mellitus, chronic renal insufficiency, and malignancies constitute the most commonly affected population [1]. Less frequently, Pyogenic spondylodiscitis affects young patients without previous comorbidities, mainly after surgical procedures. Postoperative spondylodiscitis occurs mostly after spinal surgeries or interventions [4], with a lower incidence after non-spinal procedures [6].

Only a few cases of spondylitis were reported after genitourinary procedures, including prostate biopsy [7], rectopexy [8], sacrohysteropexy [9], and vaginal delivery [10]. The pathophysiology of pyogenic spondylodiscitis may be attributed to transient bacteremia after these interventions [11,12]. Staphylococcus aureus is the most common isolated pathogen, followed by Enterobacter [13]. In 2021, a series of 40 patients with pyogenic spondylodiscitis were reported from a single center, most of them occurring after cesarean section [6]. Our case adds another example of pyogenic spondylodiscitis after genitourinary procedures without spinal anesthesia.

The differential diagnosis may also include tuberculous spondylitis [13]. However, our patient had no history of tuberculosis, no contact with an affected patient, and no history of incarceration or camp residence. Due to limited resources in the patient's country, additional cultures or GeneXpert testing were not affordable. We were able to confirm the diagnosis of bacterial spondylodiscitis based on the clinical and laboratory response after initiation of empiric antibiotic therapy.

The most common symptom of pyogenic spondylodiscitis is lower back pain, which is reported in 67-100% of cases [1]. It is important to note that lower back pain is also a common postpartum complaint due to various causes such as hormonal changes, gravity center shifts, and strained abdominal muscles [14]. Since pyogenic spondylodiscitis is a rare occurrence, the treatment may be delayed for several months due to misdiagnosis [15]. The patient in our case complained of pain for three months. Therefore, a high index of suspicion is required, and every postoperative back pain that does not respond to usual treatments should be further investigated.

Laboratory markers and cultures are essential for accurate diagnosis [1]. MRI is the imaging modality of choice. The affected vertebral bodies and disks show reduced signal on T1-weighted images and increased signal on T2-weighted images [2]. A CT-guided aspiration is indicated when the blood culture is negative, as was seen in our patient [3]. The culture of the aspirate was reported to be positive in only 48% of cases, which drops to 32% if antibiotics are already administered [16]. Our patient had already received oral antibiotics before the aspiration, which could have contributed to the negative cultures.

The treatment of pyogenic spondylodiscitis is notorious for prolonged periods of intravenous antibiotics up to 12 weeks [1]. Recently, growing evidence supports shorter treatment courses [17]. Our patient received intravenous antibiotics for two weeks: inpatient treatment for two days and thereafter as an outpatient parenteral antimicrobial therapy (OPAT), since OPAT proved to be cost-effective in bone and joint infections [18].

The treatment efficacy should be assessed by monitoring the clinical response, the inflammatory markers, and the radiological findings [1]. Our patient had dramatic improvement after aspiration, ESR dropped after treatment, and the follow-up MRI six weeks after therapy completion revealed the resolution of the pathologic findings. The resolution of the increased signal on the T2-weighted fat-saturated images reflects inflammation resolution, and the normalization of the low signal on T1-weighted images associates well with clinical recovery [2].

Surgical management can be indicated in certain situations, such as epidural or paraspinal abscesses, neurological compression, and failure to respond to medical therapy [19], which were not seen in our patients. Late complications of pyogenic spondylodiscitis include relapse and persisting back pain. Relapse occurs in 1-22% of the cases and may be seen even after years. It is important to consider relapse in any patient who presents with suggestive symptoms of spondylodiscitis with a positive previous history [20]. Our patient presented one year later with mechanical lower back pain and without systemic symptoms. The MRI revealed degenerative changes without signs of relapse, and the patient responded well to the treatment with NSAIDs.

## Conclusions

Back pain can occur after any surgical procedure, and it is mostly attributed to other causes or not taken seriously. It is important to recognize pyogenic spondylodiscitis as one of the possible causes of postoperative lower back pain after cesarean sections or genitourinary interventions, even in patients who do not receive spinal anesthesia.

When new-onset back pain occurs after surgical procedures and shows no response to the usual treatments, it should be properly investigated and never be neglected. Laboratory assessment presents the initial diagnostic measure and includes ESR, CRP, and procalcitonin. The exact level of the lesion and the extent of the disease can be localized using imaging studies, and the diagnosis is confirmed using aspiration. Long-term antibiotic therapy is the mainstay of treatment and should be administered based on clinical and radiological suspicion even if the cultures are negative.
